# Assessment of visual function using mobile Apps

**DOI:** 10.1038/s41433-024-03031-2

**Published:** 2024-03-20

**Authors:** Thaiba Bano, James S. Wolffsohn, Amy L. Sheppard

**Affiliations:** School of Optometry, College of Health and Life Sciences, Aston University, Birmingham, UK

**Keywords:** Physical examination, Outcomes research

## Abstract

With the advances in smartphone and tablet screens, as well as their processing power and software, mobile apps have been developed reporting to assess visual function. This review assessed those mobile apps that have been evaluated in the scientific literature to measure visual acuity, reading metrics, contrast sensitivity, stereoacuity, colour vision and visual fields; these constitute just a small percentage of the total number of mobile apps reporting to measure these metrics available for tablets and smartphones. In general, research suggests that most of the mobile apps evaluated can accurately mimic most traditionally paper-based tests of visual function, benefitting from more even illumination from the backlit screen and aspects such as multiple tests and versions (to minimise memorisation) being available on the same equipment. Some also utilise the in-built device sensors to monitor aspects such as working distance and screen tilt. As the consequences of incorrectly recording visual function and using this to inform clinical management are serious, clinicians must check on the validity of a mobile app before adopting it as part of clinical practice.

## Abstract (zh)

随着智能手机和平板电脑信息处理能力和软件的升级, 移动应用程序已被研发用于评估视功能。本综述评估了那些在文献中报道过的移动应用程序, 这些程序用于测量视力、阅读指标、对比敏感度、立体视力、色觉和视野, 而这些指标仅占报道用于平板电脑和智能手机指标总数的一小部分。总的来说, 研究表明, 大多数文献评估的移动应用程序都可以准确地模仿多数传统的纸质版视觉功能测试, 这得益于背光屏幕更均匀的照明, 以及同一设备提供的多个测试和版本 (用以限制病人记忆测试表) 等方面。一些设备还利用内置的传感器来监测工作距离和屏幕倾斜等。由于错误记录视觉功能并将其用于临床管理的后果严重, 因此临床医生在将其作为临床实践的一部分之前, 必须检查移动应用程序的有效性。

## Introduction

Traditional visual function tests were paper based. They were susceptible to degradation over time and with handling, it was hard to achieve even and appropriate illumination, and there was a need for multiple versions to minimise recollection bias. Software alternatives have become more common with the advent of smartphones and tablets, allowing key visual metrics to be assessed in more remote environments, but how equivalent are they to conventional testing? Technical challenges that have largely been addressed are pixel size (for near acuity optotype display), grayscale levels (for contrast testing), display size (for visual field testing) and colour rendition (for colour vision testing). On the Apple Store, a search identified over 50 apps reporting to assess visual acuity, 5 apps contrast sensitivity, 5 apps colour vision and 2 apps visual field assessment. On Google Play (Android), 13 apps reporting to assess visual acuity, 7 apps contrast sensitivity, 10 apps colour vision and 3 apps visual field assessment (January 2024). This review assesses full journal published papers validating these apps, located through a search of PubMed and Web of Science for “visual function” AND “app*” from their inception to the end of 2023, along with reviewing relevant references identified by these papers.

## Visual acuity mobile Apps

Visual acuity (VA) is defined as the ‘spatial resolving capacity’ and represents the angular size of detail that is just resolvable by the observer. Minimum recognisable resolution is the most frequently used form of VA and involves the measurement of acuity using optotypes, which take the form of letters, numerals, symbols or pictures [1]. Based on VA, the World Health Organisation [2] has estimated that over two billion people suffer from near or distance vision impairment, at least half of which is preventable or not even diagnosed. Reduced VA can lead to difficulty in performing daily tasks such as driving or working, resulting in a lower quality of life (QoL) [3] and changes in VA can be indicative of pathology or alteration in refractive error [4].

Measures of VA are important in refraction, and are usually the primary measure of visual function in the diagnosis and follow up of patients with ocular pathology, as well as providing an indication of the safety of ophthalmic products / procedures [5]. Assessment of VA may be carried out by a range of personnel and charts may be physical, projected, computerised or displayed on another screen [1, 6] Despite several limitations, the traditional Snellen chart and Snellen notation (where the numerator represents testing distance, e.g. 6 m in the UK, and the denominator is the distance in metres at which the optotype height subtends 5 min arc and the stroke width subtends 1 min arc) remain in widespread clinical use. Snellen charts have differing numbers of optotypes per line, variable progression between lines, and the notation is problematic for linear representation of visual function and subsequent statistical analysis. LogMAR (log [base 10] of the MAR [expressed in minutes]) charts, introduced in the 1970s by Bailey and Lovie [7], overcome several of the disadvantages of Snellen, with 5 letters per line (each letter having a logMAR value of 0.02), uniform inter-line and inter-letter spacing, and the ability to move the chart and scale the results for non-standard testing distances. The ETDRS (Early Treatment of Diabetic Retinopathy Study) logMAR chart is well-established for research purposes, and differs from the Bailey Lovie logMAR chart in that the letters are wider and the chart was specifically designed for a 4 m testing distance, allowing for smaller examination rooms. For the assessment of near vision, logMAR, N-point, M-scale, equivalent Snellen, and Jaeger charts are available, often comprising words or paragraphs of text, rather than individual optotypes. Near charts that follow a logMAR format offer similar advantages to logMAR distance charts, and non-logMAR near charts are often truncated meaning that many patients will not be tested to threshold [6].

Mobile apps that measure VA may confer benefits for clinicians, researchers and the wider population. Such apps may allow patients, potentially with the assistance of a carer or family member, to regularly self-monitor acuity away from the clinic, which is of value when access to services is difficult or impacted by events such as the COVID-19 pandemic; COVID-19 forced a shift towards more tele-ophthalmology services and highlighted the need for reliable and valid digital tools. In regions where healthcare services are less established, apps may be used by non-specialist healthcare workers with minimal equipment, for screening and follow-up purposes. In clinic or clinical trials, apps can provide a standardised approach to the measurement and recording of VA through their backlit screens, ability to scale the letter size based on the entered working distance, options to randomise the optotypes displayed to prevent recall bias and the potential to automate the measurement process. In non-ophthalmic healthcare settings, such as emergency rooms, mobile Apps for the assessment of VA may also be useful [8].

A summary of peer-reviewed research where the performance of commercially-available mobile Apps to measure distance (Table 1) or near VA (Table 2) has been compared with a standard clinical approach and/or employed on a large scale was tabulated. Peek Acuity (Peek Vision Ltd. https://peekvision.org/solutions/peek-acuity/), available on Android only, has been the most widely studied distance VA app to date; it uses tumbling-E optotypes, reducing the barriers of literacy/ language and age, which are associated with conventional letter-based charts. The test requires calibration to the chosen testing distance (2 or 3 m) and another individual to perform the test; studies have included diverse personnel, such as caregivers in the home, schoolteachers and healthcare workers in Africa [9–12]. The majority of users questioned have reported that the App is easy to use [10, 11]. For screening children’s vision, some earlier studies suggested the sensitivity of the App in identifying reduced vision (e.g. <6/12) required improvement before more widespread use was feasible [12, 13]. A later study in Botswana [9] used Peek in a screening programme where 16% of 12,877 children examined with the app were referred for further clinical care based on vision <6/12 in the better eye, with around half of these children confirmed subsequently as needing spectacles, ocular medication, or further clinical care. The study highlighted the potential for mobile health technologies to be employed in countries similar to Botswana for nationwide vision screening programmes.

**Table 1:** Summary of published studies that have reported on the use of mobile apps to measure distance visual acuity, in comparison to conventional clinical tests.

Reference, device and testing distance	Study population and type	Chart used in app	Comparison clinical test(s)	Key findings/ conclusions
Distance Visual Acuity				
*Peek Acuity*				
Bastawrous et al., 2015 [10] Galaxy SIII GT-19300 running Android 4.0 2 m	*n* = 300 E = 300 A = 55+	Tumbling E chart	Snellen tumbling E chart @ 3 m, ETDRS chart @4 m. At home and in office testing.	App results agreed well with clinical test providing accurate and repeatable acuity measures: 95% CI for repeatability of Peek acuity data were ±0.029 logMAR. Mean difference between Peek and ETDRS was 0.07 logMAR, and between Peek and Snellen was 0.08 logMAR. Peek Acuity test was readily accepted by Kenyan healthcare workers, taking no additional time compared to Snellen chart tumbling E test.
de Venecia et al, 2018 [13] Samsung Galaxy A3 (Android 6.0.1) 2 m	For referral agreement: *n* = 95 E = 190 A = 6–16	Tumbling E chart	Snellen chart, conducted by ophthalmologist.	Apparent overestimation of VA by Peek Acuity. 47% sensitivity and 83% specificity for identification of children with VA < 6/12, compared to an ophthalmologist using a Snellen chart. Low sensitivity could impact on paediatric screening programmes.
Rono et al., 2018 [12] Samsung Galaxy S3 2 m	*n* = 1862 E = 3724 A = 11.2 ± 2.8	Tumbling E chart	EDTRS Tumbling E	In determining visual impairment (at least one eye <6/12), sensitivity and specificity were 76.9% and 90.8%, respectively, compared to ETDRS logMAR testing. The testing algorithm requires further refinement to improve sensitivity while maintaining specificity.
Andersen et al., 2020 [60] Android phones 2 m	*n* = 12,877 A = 6–22	Tumbling E chart		App successfully used for vision screening amongst a subset of schoolchildren in Botswana, with 16% referred for optometric care due to poor vision. Low-cost portable screening tools that can be operated by novices offer potential for eye screening in other similar countries.
Satgunam et al., 2021 [61] Honor mediaPad T3 10 tablet Android version 7.0 2 m	*n* = 68 E = 68 A = 20–60	Tumbling E	COMPlog Tumbling E	Peek Acuity and COMPlog showed comparable results (on average, Peek underestimated by 1 letter), with no significant age or refractive error effects.
Bhaskaran et al, 2022 [62] Samsung Galaxy M30 Android version 10 3 m	*n* = 184 E = 360 A = 18+	Tumbling E chart	Snellen chart 6 m	VA results between Peek Acuity and Snellen chart showed no statistically significant difference. Tendency for Peek to slightly overestimate VA in eyes with reduced VA (by approximately 0.1 logMAR) App is effective, reliable, and feasible alternative to assess VA in tele-ophthalmology, especially during pandemic disruption.
Davara et al., 2022 [63]	*n* = 100 E = 100 A = 13–76	Tumbling E chart	COMPlog computerised test chart.	Good agreement between Peek Acuity and COMPlog although relatively few participants with poor acuity included. Peek Acuity can be used by caregivers to effectively measure a patient’s VA at home. 95% of caregivers felt it was easy to use. May have benefits for post-surgical monitoring.
Manasa et al., 2023 [64] Smartphone (model not stated) 3 m	*n* = 50 A = 18–25	Tumbling E chart	logMAR chart	Significant difference identified between app results and logMAR chart results in young adults.
*AAPOS Vision Screening application*				
Nik Azis et al. 2019 [65] iPad mini 3 m	*n* = 195 E = 390 A = 5–6	Lea symbols	ETDRS Lea symbols 3 m	The app shows promise as a screening tool that can be used by parents: sensitivity 86.6% (RE), 79.5% (LE), and specificity 78.9% (RE), 71.8% (LE), in identifying children with acuity worse than 0.26 logMAR, compared to optometrist examination with Lea symbols test.
Vision at Home				
Han et al., 2019 [66] iPhone 7/ 7 Plus 2 m	*n* = 163 Older Chinese (*n* = 50), Adolescent Chinese (*n* = 50), and Australian (*n* = 63) groups.	Tumbling E chart	Tumbling E ETDRS chart 4 m	Good agreement between app and ETDRS distance VA. Majority of users satisfied with use of app.
Eye Chart				
Tiraset et al., 2021 [67] iPhone X iOS 14.2 (iOS application) 4 feet	*n* = 151 E = 295 A = 18–85	Snellen (Tumbling E for those unable to read)	ETDRS 4 m	Eye Chart results corresponded well with ETDRS test showing consistency and no significant difference in measured acuity. Eye Chart has value for remote vision monitoring or may be used as a portable in-clinic test.
Bhaskaran et al., 2022 [62] iPhone SE (iOS 14.5.1) 1.2 m	*n* = 184 E = 360 A = 18+	Tumbling E	Snellen chart 6 m	No significant difference between app VA and Snellen chart results. EyeChart slightly underestimates VA compared to Snellen chart, with this tendency increasing in patients with reduced vision; differences are mainly within 0.1 logMAR. App offers a feasible alternative to assess VA in tele-ophthalmology.
Eye Chart Pro				
Hazari et al., 2022 [68] iPad Air 2 1 m	*n* = 51 (n = 26 with low vision, and n = 25 normally sighted) E = 51 A = 27–91	ETDRS chart R	ETDRS 4 m (1 m for low vision group)	No significant difference between app and clinical test results, although app shows a tendency to underestimate VA in low vison group, and overestimate in normally sighted patients. App can reliably measure VAs across a range, from normally sighted patients to those with low vision.
Smart Optometry				
Raffa et al., 2022 [69] iPhone 11 and Honor play (Android)	*n* = 100 E = 200 A = 5–16	Tumbling E	Snellen chart Tumbling E 6 m	Mean difference between Snellen and Smart Optometry VA was −0.023 logMAR (1 letter). App detected subnormal vision (worse than 20/30) with sensitivity of 89.3% and specificity of 69.4%; and detected amblyopia (≥2 line difference between eyes) with sensitivity of 58.3% and specificity of 83%. Improvements in sensitivity are needed before the tool could be used in broader telehealth programmes involving children at risk of amblyopia.
Trec Oculistica				
Racano et al., 2023 [70] Android and iOS phones 3 m	*n* = 41 E = 82 A = 4.4–6.1	Snellen	LEA Symbols Snellen chart	App VA results were moderately congruent with clinical testing. For home testing, some families found the protocol complicated, and some children were un-cooperative (N.B. young age of cohort).
*K-VA*				
Karampatakis et al., 2023 [71] Samsung A30S Android OS (5.0) 1 m	*n* = 171 E = 250 A = 18–89	Sloan letter chart	ETDRS 4 m ETDRS near VA card – 40 cm	App measurement taken by participants; 73.2% reported that the test was easy to use. Mean difference between Keratopathy-VA at 1 m and ETDRS at 4 m was −0.006 logMAR. High test-retest repeatability. May be a useful tool for patients who struggle to access healthcare services, or for following up those with accessibility issues.

*n* = number of participants; E = number of eyes included; A = age of participants (years).

**Table 2:** Summary of published studies that have reported on the use of mobile apps to measure near visual acuity, in comparison to conventional clinical tests.

Near Visual Acuity				
*Smart Optometry*				
Satgunam et al. 2021 [61] Honor mediaPad T3 10 tablet (Android version 7.0) 40 cm	*n* = 68 E = 68 A = 20–60	Tumbling E	Reduced Snellen near vision chart – Tumbling E	Significant difference was found between the app and clinical test results with the App. SmartOptometry may correlate better with Reduced Snellen VA if a patient’s acuity is poor and not when it is good, due to pixelation of digital screens.
Raffa et al., 2022 [69] iPhone 11 and Honor play (Android)	*n* = 100 E = 200 A = 5–16	Tumbling E	Rosenbaum pocket vision screener Tumbling E	Small but significant difference in near VA with App and near chart. App underestimated VA by up to 2 letters typically.
*Vision at Home*				
Han et al., 2019 [66] iPhone 7/ 7 Plus 40 cm	*n* = 163 Older Chinese (*n* = 50), Adolescent Chinese (*n* = 50), and Australian (*n* = 63) groups.	Tumbling E	Tumbling E ETDRS near card.	App results comparable to ETDRS near card (typically <1 line difference), across all groups. Majority of users satisfied with use of app.
*Peek Near Vision (PeekNV)*				
Katibeh et al., 2022 [72] Sony Xperia 10 II	*n* = 483 E = 483	Tumbling E	Tumbling E near point vision chart	Mean difference between PeekNV and chart NVA results was 0.008 logMAR. Participants were patients at an outpatient eye clinic in Nepal. For screening patients with inability to read N6 at 40 cm, the positive predictive value of PeekNV was 93.2%, and the negative predictive value 92.7% App may be useful in screening populations at risk of presbyopia.
*K-VA*				
Karampatakis et al., 2023 [71] Samsung A30S (Android OS 5.0) 40 cm	*n* = 171 E = 250 A = 18–89	Based on ETDRS, with some different letters.	ETDRS near VA card – 40 cm	Agreement between K-VA test at 40 cm and near ETDRS chart was high at −0.007 (95% LoA −0.105 to 0.090) logMAR. Test–retest repeatability showed mean difference of 0.005 logMAR for App at 40 cm.
*New York University (NYU) Lagone Eye Test*				
Iskander et al., 2022 [73] iPhone 8 (iOS 14.3) Motorola Moto x4 (Android version 9) 14 inches	*n* = 125 E = 244 A = > 18	Number-matching	Rosenbaum near card 14 inches	Correlation between App and Rosenbaum near card was r = 0.74 for both iPhone and Android. Test–retest reliability was 0.003 ± 0.22 logMAR (iPhone), 0.01 ± 0.25 logMAR (Android), and 0.01 ± 0.23 logMAR (Rosenbaum card). 97.6% of participants found the App easy to use.
*Sightbook*				
Kim et al. 2022 [74] iOS devices 36 cm	*n* = 200, with a range of eye conditions. E = 200 A = 59.9 ± 15.4	Snellen-style	Good-Lite near-vision chart 35 cm, performed in-clinc by technician.	Correlation between Sightbook and Good-Lite NVA was r = 0.880; *P* < 0.001 No significant differences in measured VA between App and Good-Lite in any of the groups (normal, cataract, ARMD, diabetic retinopathy). Further validation required, but App shows promise for remote monitoring of patients,

*n* = number of participants; E = number of eyes included; A = age of participants (years).

A 2022 meta-analysis [14] of the performance of mobile apps for VA assessment reported that when such apps were used by non-professionals, the accuracy was better than for professionals, a finding that was attributed to adults such as parents or schoolteachers having a better understanding of children’s responses, behaviour and moods than eye care professionals who are not known to the children being examined. The age of participants may also impact on the results obtained, with the sensitivity and diagnostic odds ratios of mobile VA apps being significantly better when adults are examined rather than young children. Overall, the body of literature to date indicates that mobile apps for the assessment of VA can be used successfully by professionals and non-professionals, in non-clinical settings, and that the apps generally perform well. Further research is needed, to include a wide range of participant ages and levels of vision, but the apps offer significant potential for the assessment and follow up of patients receiving ophthalmic care, and for children’s vision screening, especially in low-income countries.

## Reading metrics

While high contrast, static VA is an important safety and disease detection metric, [15] it is not a good predictor of functional vision. [16] Most near tasks involve an element of reading, [17] which is perceived as being critical to communication and commerce in modern societies. [18] The speed at which an individual reads is fairly consistent until the critical print size is reached, after which it rapidly slows until the individual is no longer able to differentiate the optotypes (the near acuity threshold). [16, 19] Paper based reading speed charts were developed in the 1990s, [20–22] with the time to read paragraphs of text that sequentially decreased in size out aloud, manually timed; the results then had to be plotted to determine the supra-threshold reading speed, critical print size and near threshold, which was time consuming [23] A study comparing a digitised version of the MNRead chart found the results were similar, although the reading speed was slower on a tablet, attributed to the different method of timing the reading trials [24].

While the text and sizing can easily be digitised, using the onboard sensors allows for the working distance to be monitored through the camera, the start and end of the reading of each paragraph to be accurately timed through the microphone as well as recording to allow for incorrect syllable detection, and immediate data analysis. This approach was used when digitising the Radner chart, demonstrating a faster reading speed and lower critical print size when using the tablet app, and equivalent to better repeatability than the equivalent paper based version [25].

A novel Greek reading speed app (GDRS-test) on an Android device consisting of the time to read aloud (at a 40 cm distance) a series of 30 random two-syllable and then 30 three-syllable Greek words at the critical print size, without semantic connection, showed a moderate correlation between correct words per minute and the MNRead Chart assessed reading speed [26] However, the critical print size needed to be calculated in advance.

## Contrast sensitivity

Conventional contrast sensitivity tests measure contrast detection at one spatial frequency [27], or spatial frequency at two to nine contrasts [28, 29]. A digital version of the Pelli-Robson chart, consisting of three letters for each contrast level (LogCS range of 0.15–2.25 with 0.15 LogCS steps) viewed at 80 cm was found to be more accurate and have a wider range of contrast stimuli than the paper chart, as well as the ability to assess both positive and negative polarity [30]. However, the OdySight digital version of the Pelli-Robson chart underestimated the contrast sensitivity by 0.16 logCS and the limits of agreement were large, showing it to be unreliable [31]. The Peek approach to contrast sensitivity presents a single tumbing ‘E’ at 1 m in one of four directions starting at maximum contrast and the user points in the direction of the prongs and the examiner ‘swipes’ the letter in that direction; if correct the contrast is reduced, with two incorrect direction swipes being the endpoint. This approach was highly correlated to a tumbling ‘E’ version of the Pelli-Robson chart, taking a faster but  statistically similar time to complete [32]. Another study measured contrast sensitivity with sinusoidal gratings of 3, 6, 12 and 18 cycles per degree on a tablet compared to the similar Functional Acuity Contract Test (FACT) included in the Optec 6500, finding similar results [33].

Robson and Campbell originally portrayed the contrast sensitivity function as a sine wave grating with varying contrast (Y-axis) and spatial frequency (X-axis) [29]. Using a bit-stealing method, this can now be displayed on a tablet screen, with the user tracing their finger where they can see the tops of the maxima [34]; this approach showed much greater repeatability than CSV-1000 contrast test; Pelli-Robson had the best repeatability, but only assessed one spatial frequency as opposed to the app which generated a complete contrast sensitivity function in under a minute. It has been suggested that generating a curve in this way is not accurate, but instead of tracing the curve, the authors fitted their own simulation of the sinusoidal function with only 4 points [35].

## Stereoacuity

A small number of stereoacuity mobile apps are available and have been evaluated in the peer-reviewed literature. The Tablet Stereo Test (TST) is an iPad-based app for the assessment of stereoacuity, based on random dot stereograms, viewed through anaglyph glasses (with red and green/blue filters to separate the images between the eyes). The observer is required to indicate the direction of the missing segment of a circle (one of four directions) and the test can be performed at multiple distances. Compared with the TNO test for near stereoacuity used at 3 m and 50 cm, there was no significant difference in median values between the app and the clinical test in adult participants [36]. The Android application SAT, also based on anaglyphs, has options to display random dot image sets including TNO, LANG, LEA, LEA contours, letters, and Pacman, taking 45–60 s to determine a staircase algorithm threshold. Despite the longer testing time, a cohort of 497 children aged 6–11 years were all able to complete testing with the app. However, the thresholds obtained from the app were statistically dissimilar to those found with conventional TNO and Weiss EKW tests, but clinically similar, and the correlations between the tests were only moderate (r = 0.49–0.53) [37].

## Colour vision

Colour rendering on iPhones is considered sufficient for clinical assessment, although the five Ishihara apps (with varying number of Ishihara plates displayed) were found to vary in their colour accuracy [38] Although under simulated vision loss it has been suggested that the effect on colour vision might be underestimated [39], a comparison on an iPhone and a Samsung (Android) phone of the Eye Handbook colour vision test found no significant difference with a paper version [40] and a 92% sensitivity and 100% specificity [41]. However, while the Eye2Phone Ishihara test was found to have a high sensitivity (100%), specificity (95%) and coefficient of agreement (r = 0.95), the Colour Vision Test app was much poorer (100% sensitivity, 55% specificity and coefficient of agreement r = 0.535) [42]. Displaying the test on a Liquid Crystal Display (LCD) monitor also resulted in a 100% sensitivity and 99% specificity [43].

A comparison between paper and tablet displayed Velhagen/Broschmann/Kuchenbecker colour plates found 83% coincidence in findings in those with colour vision deficiencies and 89% in those considered colour normal [44] An app of the Farnsworth-Munsell Hue-100 cap ordering test compared to the original analogue version found poor comparability of results [45] A web-based Colour Assessment and Diagnostic test from City University (which uses random luminance masking) showed a high sensitivity (93–100%), specificity (83–100%) and coefficient of agreement (0.83–0.96) compared to the Nagel anomaloscope “gold standard”, Ishihara and the FM-100 hue, although the HRR only had a specificity of 33% and a coefficient of repeatability of 0.33 [46].

A novel gamified tablet-based ColourSpot test requires children, as young as four, to tap the spot in the grey background ‘sky’ to reveal an animation. Each target type (protan, deutan, tritan) is presented at high saturation and if successfully identified, its saturation is multiplied by a factor of 0.5 for the next trial, thus decreasing the saturation of targets of that type; if a distractor is tapped, the saturations of all three target types are multiplied by a factor of 1.5 for the next trial, thus making the next trial easier. This approach achieved a 100% sensitivity and 97% specificity for classifying a colour vision defect compared to the Ishihara [47].

## Visual field assessment

Smartphones can be mounted in head-mounted visors to allow targets to be presented across approximately a 30° visual field. While this coverage is insufficient to detect peripheral defects, it has been established that only 1–2% of defects that are not glaucomatous have abnormalities beyond 30° without an additional central defect [48] Smartphone-based campimetry (Sb-C) [49] has been used to simulate a 59 test position threshold Octopus G1 programme showing a high (r = 0.815) correlation and moderate (r = 0.591) re-test reliability. “Visual Fields Easy” (VFE) is a suprathreshold iPad application, which when compared against the Humphrey Frequency Doubling Technology N-30-5, took on average 2.4 min longer, and only had a sensitivity of 67% and specificity of 77% [50]. Other studies compared the VFE with the Humphrey SITA Fast 24-2, finding sensitivity (78/90/97%) and specificity (53/48/70%) increased with glaucoma severity from mild to moderate to severe (respectively) [51, 52]. Another approach has been the Melbourne Rapid Fields (MRF) with a radial test pattern comprised of 66 test locations [53], which was similar in speed to the Humphrey 24-2 SITA fast and faster than SITA standard, was highly repeatable and had an inter-class correlation coefficient of 0.71–0.93 [54, 55].

As part of the Vision Impairment Screening Assessment (VISA) Stroke Vision app, the dynamic visual field test involves the user viewing a red fixation cross and tapping the screen when they detect a black target moving towards this fixation point [56]; this demonstrated a 79% sensitivity and 88% specificity compared to Goldmann or Octopus kinetic perimetry [57] and 0.70 Kappa agreement with confrontation fields [58].

As part of Read-Right post-stroke therapy, their app binocularly assesses visual fields with an adaptive algorithm testing of six points in each hemi-field, at 1, 2.5, 5 and 10° on the horizontal meridian and two additional points above and below the horizontal meridian at 2.5°; points are displayed for 100 ms at 5 dB suprathreshold, with the points closer to fixation having reduced contrast. Compared with Humphrey 10-2 and 24-2 perimetry in patients with unilateral homonymous visual field defects, the sensitivity (≥79%) and specificity (≥75%) for points along the horizontal meridian were best [59].

## Conclusions

Mobile apps can mimic most traditionally paper-based tests of visual function. They can also benefit from more even illumination from the backlit screen, randomisation, and use of the in-built sensors to monitor aspects such as working distance and screen tilt. These features are particularly important when home assessment is advocated. However, this review only assessed those apps that have been evaluated in the scientific literature which constitute just a small percentage of the total number of apps available for tablets and smartphones. The consequences of incorrectly recorded visual function and using this to inform clinical management are serious, and therefore clinicians must check on the validity of a mobile app before adopting it as part of clinical practice.
